# Profiles of cardiometabolic risk and acculturation indicators among South Asians in the US: latent class analysis of the MASALA study

**DOI:** 10.3389/fpubh.2024.1384607

**Published:** 2024-10-08

**Authors:** Francisco A. Montiel Ishino, Katia M. Canenguez, Jeffrey H. Cohen, Blake Victor Kent, Kevin Villalobos, Belinda L. Needham, Namratha R. Kandula, Alka M. Kanaya, Alexandra E. Shields, Faustine Williams

**Affiliations:** ^1^Division of Intramural Research, National Institute on Minority Health and Health Disparities, National Institutes of Health, Bethesda, MD, United States; ^2^Division of Intramural Research, National Institute of Environmental Health Sciences, National Institutes of Health, Durham, NC, United States; ^3^Harvard/Massachusetts General Hospital Center on Genomics, Vulnerable Populations, and Health Disparities, Boston, MA, United States; ^4^Department of Anthropology, The Ohio State University, Columbus, OH, United States; ^5^Department of Sociology, Westmont College, Santa Barbara, CA, United States; ^6^Department of Epidemiology, University of Michigan, Ann Arbor, MI, United States; ^7^Institute for Public Health and Medicine - Center for Community Health, Northwestern University Feinberg School of Medicine, Chicago, IL, United States; ^8^Department of Medicine, Northwestern University Feinberg School of Medicine, Chicago, IL, United States; ^9^Departments of Medicine, Epidemiology, and Biostatistics, University of California, San Francisco, San Francisco, CA, United States

**Keywords:** cardiometabolic disease, South Asian, latent class analysis, social determinants of health, acculturation

## Abstract

**Background:**

South Asians (SA) represent the fastest growing US immigrant group, and previous studies have indicated that they face disproportionately high burden of cardiometabolic disease. Cardiometabolic disease manifests as a syndemic or synergistic epidemic encompassing multiple disease clusters influenced by biological, social, and psychological factors stemming from the acculturative process. This process may exacerbate morbidity within immigrant subgroups. Our aim was to identify cardiometabolic risk profiles among SA using indicators of acculturation.

**Methods:**

We conducted a latent class analysis on data from the Mediators of Atherosclerosis in South Asians Living in America study (*N*=771). A composite cardiometabolic disease outcome was constructed using prevalent hypertension, type 2 diabetes, and body mass index. Acculturation indicators included years living in the US, English language proficiency, dietary behaviors, preservation of cultural traditions, social and neighborhood support, maintenance of social relationships (i.e., friendships), and experiences of discrimination, along with proxies of acculturative stress (i.e., depressive symptomology, trait anxiety and anger). Social and environmental determinants of health, health behaviors, religiosity and spirituality served as covariates to further assess latent class membership.

**Results:**

Four cardiometabolic risk profiles emerged: (1) lowest risk [73.8% of sample] characterized by high integration into both SA and US cultures; (2) the modest risk [13.4% of sample], exhibiting elevated levels of mental health distress and experiences of discrimination, and distancing themselves from both cultures; and the (3) moderate risk [8.9% of sample] and (4) highest risk [3.9% of sample], demonstrating greater assimilation into US culture. Compared to the lowest risk profile: the modest risk profile was associated with low-income and conflicting attitudes about religion/spirituality, while the moderate risk profile was characterized by lower income and educational attainment with positive behaviors and attitudes toward religion/spirituality.

**Conclusion:**

Findings expand our understanding of immigrant cardiometabolic health as a syndemic issue wherein multiple co-occurring and interacting processes synergize to produce negative outcomes in already at-risk subpopulations. Furthermore, acculturation emerges as a crucial factor in understanding health disparities among immigrant and refugee groups in the US.

## Introduction

Asians are currently the fastest growing immigrant group in the United States (US), after Hispanic/Latino groups (1). Among Asians, those categorized as South Asian (i.e., individuals from India, Pakistan, Bangladesh, Sri Lanka, Nepal, and Bhutan) are faster growing and have been reported to have a high incidence and prevalence of atherosclerosis, in addition to higher rates of myocardial infarction, coronary artery disease, and stroke events (1, 2). South Asians face an increased risk of cardiometabolic disorders such as hypertension, type 2 diabetes, and higher adiposity (3, 4). In South Asian countries, hypertension prevalence among all adults ranges from 31 to 45% compared to 29% in the US (5); and type 2 diabetes prevalence ranges from 11 to 24% compared to 8.9% in the US (6). In a United Kingdom study, the onset of type 2 diabetes and heart disease was found to occur 5–10 years earlier in South Asians when compared to their White counterparts (7, 8).

With the extant epidemiological data and existing interventional efforts to manage cardiometabolic conditions like type-2 diabetes, reports for successful programs vary among South Asians communities globally (9, 10). Cardiometabolic disease is a complex syndrome that is underexplored among US South Asian immigrants. Moreover, cardiometabolic syndrome is linked with atherosclerotic burden (11), in addition to the synergy of chronic conditions and outcomes interacting with social, psychological, and environmental factors that foment a syndemic or synergistic epidemic (12). As such, to understand this cardiometabolic syndemic, a transdisciplinary approach must be used to understand the complexities of cardiometabolic health holistically among the underserved, underrepresented, and hard-to-reach US South Asians. The holistic view of South Asian health integrates the spiritual, psychosocial, and physical, as well as the biomedical (13, 14). Our goal then is to identify profiles of cardiometabolic risk based on the immigrant experience of acculturation and acculturative stress. In identifying these cardiometabolic profiles, we can best explore known and latent risk profiles using the available yet limited data on US South Asians. As such, we can meaningfully address the health disparity, as well as rapidly assess risk and develop interventional programs that can be tailored by encompassing not only biomedical risk factors but the social, cultural, and environmental influences on promoting health behaviors. It is of critical public health importance to embrace a broader perspective beyond biomedical models to saliently address the syndemic nature of cardiometabolic disease among US South Asians, acknowledging social, environmental, and acculturative factors that impact health outcomes (15).

### Acculturation and the social and environmental determinants of health

Social and environmental determinants of health, including discrimination and structural racism perpetuated by social and economic policies, significantly impact morbidity and mortality rates, particularly among marginalized populations (16, 17). Despite their importance in health disparities research, these determinants are often overlooked in studies concerning immigrant populations and acculturation processes (16–18).Additionally, environmental factors, such as neighborhood dynamics, are absent from acculturation frameworks. To fully understand the role of acculturation, it is essential to recognize the environment as an adaptive context for immigrants, where place-related risk exposures shape health outcomes (18).

Although social and environmental determinants alone may not strongly predict cardiometabolic disorders among South Asians in the US (2, 19), they intersect with societal and ecological factors like social support and neighborhood environment. For instance, low social cohesion among US South Asian women correlates with higher BMI, while living in neighborhoods with high social cohesion reduces the odds of hypertension (20). Social networks also influence physical activity and body image norms among South Asians, impacting health-related discussions and emotional closeness (19, 21–25). Acculturation, integral to understanding South Asian health, encompasses these determinants as immigrants navigate a new cultural landscape (24–27).

### Acculturation, acculturative stress, and mental health

Acculturation involves numerous psychosocial stressors as individuals adapt to a new culture, resulting in acculturative stress (28–30). Acculturative stress refers to the psychosocial stressors experienced by individuals as they navigate and adapt to the host culture (30). This stress, marked by depressive symptoms and varying levels of anxiety and anger (29), has been extensively studied in Hispanic/Latino immigrant groups. In the MASALA study, Needham et al. (31) identified three acculturation strategies: separation (preference for South Asian culture), assimilation (preference for US culture), and integration (balanced preference for both cultures). Even with adaptive strategies, acculturation remains stressful, leading to psychological distress and exacerbating mental health symptoms. For example, MASALA participants in the separation group reported more depression than those in the integration group (31).

Acculturative stress is multifaceted, arising from interactions between individuals and the host culture (26, 30, 32). These interactions can have positive effects, such as improved well-being from maintaining ethnic identity, or negative effects, such as the detrimental impact of discrimination on physical and mental health (30, 32–37). Understanding acculturation’s complexity, including its stress and resilience aspects, is crucial for developing effective prevention and intervention programs for cardiometabolic health among immigrants. Researchers need to consider psychological, environmental, and acculturative factors to address the unique challenges faced by US South Asians, ultimately fostering a holistic understanding of how acculturative strategies impact both psychological and physiological health.

### Acculturation and cardiometabolic health

The relationship between acculturation and cardiometabolic risk among South Asians is less studied than among Hispanic/Latino immigrants, yet notable associations exist. Acculturation among South Asians has been linked with cardiometabolic disorders (1, 25). For example, longer residence in the US correlates with higher coronary artery calcium levels, while adherence to moderate traditional cultural beliefs associates with lower common carotid intima-media thickness (1). Acculturation, length of residence, and dietary patterns also impact cardiometabolic risks in US South Asians (38, 39). Traditional beliefs influence diet, with those adhering to traditional practices more likely to consume sweets, fried foods, and high-fat dairy, and less likely to eat animal protein (39). Diets high in animal protein, fried snacks, sweets, and high-fat dairy correlate with adverse metabolic risk factors, whereas diets rich in fruits, vegetables, nuts, and legumes are linked to lower hypertension and metabolic syndrome prevalence (40). Both Western and vegetarian dietary patterns were have also been associated with some metabolic risk among US South Asians (41). Thus, diet reflects an adaptation between heritage and host cultures (39, 42). To understand the syndemic nature of cardiometabolic disease among South Asians, it is crucial to consider various acculturation dimensions such as length of stay, linguistic patterns, cultural preservation, social networks, and neighborhood environment (31). These factors at social and environmental levels can synergize to influence health outcomes.

### Acculturation and religiosity/spirituality

Religiosity and spirituality (R/S) are often overlooked in acculturative measures, despite empirical associations with mental health, cardiometabolic outcomes, and stress coping mechanisms. Few studies have examined coping strategies and behaviors alongside psychosocial stressors and acculturative stress (43, 44), yet R/S coping strategies may shed light on the acculturation process by offering adaptive social and environmental mechanism for immigrants to make sense of their circumstances (45). For instance, negative R/S coping (indicative of religious struggle) has been linked to greater anxiety and anger, while positive R/S coping associated with gratitude has been linked to lower levels of depressive symptoms (46). Notably, US South Asians are less likely than other US groups to say they use R/S to cope with stress, with 48% saying they do this a great deal compared to 58% of whites, 65% of Hispanic/Latinos, and 76% of Blacks (47). Other religious/spiritual behaviors and attitudes, such as congregational attendance and closeness to God or the divine, play an instrumental role in immigrant and refugee health (48–50). Moreover, individual R/S behaviors and attitudes strongly predict for self-rated health, health biomarkers, disease, and BMI (51, 52). Among the South Asian traditions like Hinduism and Islam, for example, lipoprotein and lipid levels were found to be differently associated while accounting for multiple R/S and lifestyle factors (53). Across all South Asians, gratitude, feelings of connection to all life, and closeness to God were associated with self-rated health (46). Within certain South Asian religious groups (e.g., Sikhs; Hindus), however, an increase in self-identified R/S was associated with an increased likelihood of overweight and obese BMI (52). Furthermore, R/S can serve as an effective proxy to understand and approach mental health, which may be taboo among immigrant groups (45, 46, 54). For instance, South Asian R/S has been associated with mental health in addition to metabolic issues (46, 53). Overall, R/S behaviors and attitudes can provide a nuanced understanding of external socioeconomic and sociopolitical interactions, while R/S coping strategies can help explain internal mental health processes among immigrants (46, 51). Both R/S facets can provide context for interactions and integrations into a host culture (44) potentially differentiating individual diseases within- and between-persons.

### The present study

The role of acculturation in influencing cardiometabolic risk among South Asians remains underexplored. Syndemic theory provides a holistic framework to understand the interplay between dynamic acculturation and cardiometabolic risk in this population, but current understanding is limited. To address this gap, we first employed a person-centered approach within a syndemic framework to identify to identify cardiometabolic risk profiles based on hypertension, diabetes, and obesity probabilities, considering acculturation and acculturative stress proxies. We then examined how socioenvironmental determinants, health behaviors, and religious/spiritual behaviors and attitudes differentiate these risk profiles. After identifying cardiometabolic risk profiles and examining non-acculturation specific covariates, we discussed potential acculturation strategies for each profile. Our aim was to build on the acculturation constructs developed by Kanaya et al. (1) and Needham et al. (31), integrating models and strategies from Berry (26, 27, 32) and LaFromboise et al. (33) to refine our understanding of the complex and dynamic acculturative processes affecting cardiometabolic risk among South Asians.

## Materials and methods

### Mediators of atherosclerosis in South Asians living in America cohort study sample

The MASALA study is an ongoing longitudinal community-based cohort designed to understand heart disease and associated non-conventional risk factors to better inform prevention and tailor interventions. The MASALA study has collected multiple qualitative and quantitative data points associated with physiological health and mental health, as well as religious/spiritual beliefs and practices. R/S data were collected through the Study on Stress, Spirituality and Health (SSSH) during a MASALA study follow-up visit. Our sample consisted of adults between the ages of 40–84 that completed Exam 1 of the MASALA study and the SSSH supplement between October 2010 to March 2013. Participants were of South Asian descent from India, Sri Lanka, Nepal, Bangladesh, and Pakistan. Study participants were recruited from the San Francisco Bay and greater Chicago areas, had to be free of cardiovascular disease, and able to speak, read, and write in English, Hindi, or Urdu. See Kanaya et al. (55) for further detail on MASALA study objectives, methods, and cohort description. The MASALA study was approved by Institutional Review Boards at University of California San Francisco (#10–00353) and Northwestern University (#STU00019837). Data for our study were acquired on 15 February 2020 via application process to the MASALA program. No human subjects were involved in our study as data received and used for our analyses were secondary in nature and included no personal nor identifiable information. Therefore, no review was conducted by an Institutional Review Board for this study. Data used for this analysis are restricted but can be made available upon request to the MASALA program via application process.^1^

### Latent class analysis

We conducted a latent class analysis (LCA) using Mplus version 8.4 (Muthén & Muthén). The distal outcome of cardiometabolic risk was assessed using three observed variables: (1) hypertension based on National Cholesterol Education Program criterion [systolic blood pressure of >140 mm Hg or diastolic blood pressure of >90 mm Hg and/or medication use for hypertension]; (2) diabetes based on glucose tolerance with plasma glucose or serum glucose [i.e., classified by fasting glucose ≥126 mg/dL, and/or 2-h post-challenge glucose ≥200 mg/dL, or use of a diabetes medication]; and (3) body mass index (BMI) based on Asian categories [normal <23.0 kg/m^2^; overweight 23.0–27.5 kg/m^2^; and obese ≥27.5 kg/m^2^] (56). To contextually model cardiometabolic risk profiles among our sample of South Asians living in the US, we used a syndemic approach to holistically capture the acculturative process and associated stress. We used the following indicators to capture the acculturative process: (1) years living in the US [0–10 years; 10–20 years; or more than 20 years]; (2) spoken English proficiency [not at all or poorly; fairly well; or well or very well]; (3) food eaten at home [only or mostly South Asian; equally South Asian or other; and mostly or only other]; (4) desire to pass down cultural traditions [little to none; some; or high desire]; (5) similarity of peer groups to self [only or mostly South Asian; equally South Asian or other; and mostly or only other]; (6) social support [little to none; some of the time; or most to all of the time]; (7) neighborhood support [little to none; or most to all of the time]; (8) discrimination using a composite score from the Everyday Discrimination Scale (57) [never to rarely; or weekly to daily]; (9) religious/spiritual struggles continuous scores; and (10) positive spiritual coping continuous scores. Proxies of acculturative stress in our syndemic model included the following mental health indicators: (1) depressive symptoms using the 15-item Center for Epidemiological Studies for Depression or CES-D scale scores (58); and (2) trait anxiety and (3) trait anger using 10-item Spielberger Trait Anxiety Inventory scale or STAXI scores (59). CES-D scores range from 0 to 60, where 16 or greater indicates depressive symptoms (58). STAXI scale scores range from 10 to 40, where scores of 10–14, 15–21, and 22–40 indicate low, moderate, and high levels of trait anxiety or anger (59, 60).

### Covariates

**Figure 1:**
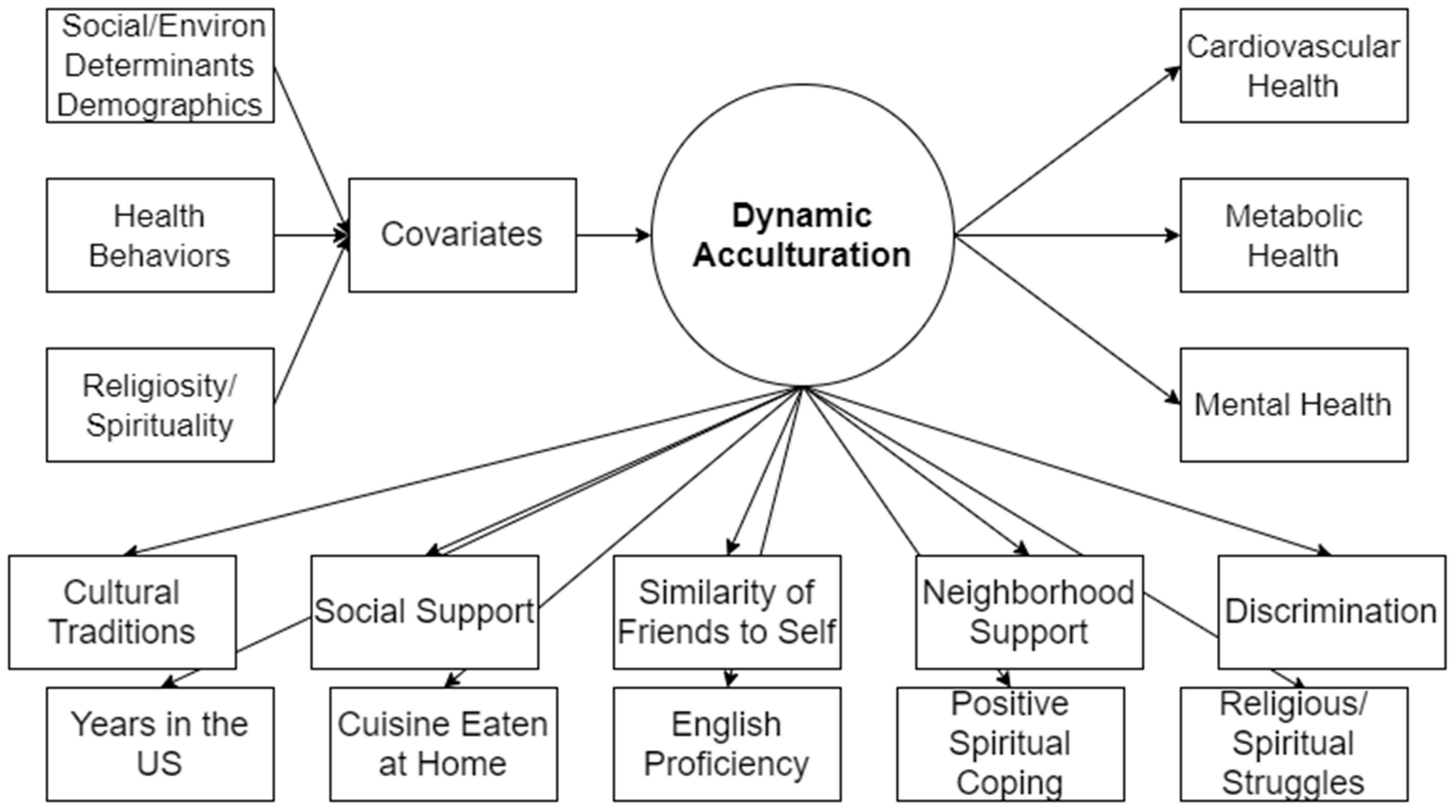
Latent class analysis model.

Sociodemographic factors including social and environmental determinants of health, health behaviors, and R/S were used as covariates to further differentiate latent class membership. *Sociodemographic factors including social and environmental determinants of health.* Age [under 54 years of age = 0; or 55 years of age or older = 1], annual household income [income under $75,000; or income of $75,000 or above], education [less than a bachelor’s degree = 0; or bachelor’s or higher = 1], and geographic place of residence [San Francisco = 0; or Chicago = 1]. *Health behaviors.* Self-reported physical activity defined as nonessential activities of daily living in the last 12 months on “how often did you exercise?” [low to inactive based on response of “never” or “a few times a year” = 0; or moderate or higher levels based on a response of “monthly,” “weekly,” “almost/daily” = 1]; yoga or take part in spiritual, mental, physical, or meditative techniques rooted in South Asian philosophical traditions [never = 0; or any frequency based on a response of “a few times a year,” “monthly,” “weekly,” “almost/daily” = 1]; smoking tobacco [former and current smoker = 1; or never smoker = 0]; alcohol use [no alcohol use = 0; or more than 1 or more drink(s) per week = 1]; and does participant eat out often [less than once a week = 0; or once a week or more = 1]. *Religiosity and spirituality behaviors and attitudes.* Religious or spiritual tradition [Hindu religious traditions = 1; or all others including Muslim, Jain, Sikh, Zoroastrian, Judaism, Christian, other religious traditions, multiple traditions, and agnostic/atheist = 0], find it difficult to forgive myself for things I have done [disagree or strongly disagree = 0; or strongly agree or agree = 1], feeling that others have not forgiven me for things that I have done [disagree or strongly disagree = 0; or strongly agree or agree = 1], inner peace [never = 0; or any frequency = 1], spiritual connection to all life [less than daily = 0; or many times a day to every day = 1], spirituality is about their connection to [God] [strongly or somewhat agree = 1; or all other responses = 0], gratitude expressed to God [definitely or tends to be true = 1; or all other responses = 0], and frequency of religious attendance [once a week or more = 1; or never to once a month = 0] (see Figure 1 for detailed model).

### Latent class analytic plan and model fit criteria

Our latent class analysis (LCA) employed a comparative approach to identify the optimal model based on the number of classes or profiles. Multiple models from a one-class solution onward were compared by model fit indices that included Bayesian information criterion (BIC), sample-size-adjusted-BIC (ssaBIC), Bootstrap Likelihood Ratio test (BLRT), and entropy or the quality of classification. These indices were used to compare and assess model fit, while taking into consideration both practical and theoretical considerations of each model (61). Once the final model was selected, an auxiliary multinomial logistic regression was conducted to examine the role of covariates not initially included in the LCA. The auxiliary analysis aimed to further distinguish between the identified latent classes and predict latent class membership. Analytical files are available upon request.

## Results

The South Asian sample consisted of primarily of males (55.9%) who identified as Hindu (61.7%) and reported an income over $75,000 (76.1%), along with educational attainment at or above a bachelor’s degree (89.4%). The mean Spielberger trait anxiety and anger scores were 15.9 each, while the mean CES-D score was 7.3. The mean score for religious/spiritual struggles was 1.4, and for positive spiritual coping it was 2.7. The prevalence of hypertension and diabetes in this sample was 49.2 and 24.1%, respectively, with a mean BMI of 25.9 kg/m^2^. Further details can be found in Table 1.

**Table 1:** Mediators of atherosclerosis in South Asians living in America study sample descriptives (*N* = 771).

	N	%
Age category (R:40, 81)		
Under 54	391	50.7
55 and older	380	49.3
Sex		
Male	431	55.9
Female	340	44.1
Religious/spiritual tradition		
Hindu	445	61.7
Muslim	33	5.8
Jain	35	4.9
Sikh	51	7.1
Other	26	3.6
Multiple	55	7.6
None	76	10.5
Income		
Under $75,000	179	23.9
$75,000 and over	570	76.1
Level education		
Less than bachelor’s	82	10.6
Bachelor’s and above	689	89.4
Hypertension		
No	392	50.8
Yes	379	49.2
Diabetes status		
None	330	42.8
Pre-diabetes	255	33.1
Diabetes	186	24.1

	Mean	Variance
Spielberger trait anxiety scale	15.9	18.3
CES-D scale	7.3	45.4
Spielberger trait anger scale	15.9	13.8
Religious/spiritual struggles (R:1,4)	1.4	0.3
Positive spiritual coping (R:1,4)	2.7	0.8
BMI Asian category	25.9	15.8

As shown in Table 2, the majority of the sample reported residing in the US for over 20 years (69.5%), speaking English well or very well (88.2%), predominately consuming South Asian cuisine at home (52.8%), expressing a desire to pass down cultural traditions (55.6%), experiencing frequent social support (75.2%), and neighborhood support (80.4%), having predominately South Asian friends (54.1%), and experiencing discrimination on a daily to weekly basis (81.2%).

**Table 2:** Acculturation descriptives of mediators of Atherosclerosis in South Asians living in America study participants (*N* = 771).

	N	%
Years in the US		
0–10 years	45	6.0
10–20 years	184	24.5
Over 20 years	523	69.5
Speaking English		
Not at all or poorly	24	3.1
Fairly well	67	8.7
Well or very well	680	88.2
Food eaten at home		
Only or mostly South Asian	407	52.8
Equally South Asian or other	307	39.8
Mostly or only other	57	7.4
Desire to pass down cultural traditions		
Little to none	143	18.6
Some	428	55.6
High	199	25.8
Social support		
Little to none	46	6.0
Some of the time	145	18.8
Most to all of the time	580	75.2
Neighborhood support		
Little to none	151	19.6
Most to all of the time	620	80.4
Friends		
Only or mostly South Asian	417	54.1
Equally South Asian or other	276	35.8
Mostly or only other	78	10.1
Discrimination		
Rarely to never	145	18.8
Daily to weekly	626	81.2

### Latent class analysis findings

**Figure 2:**
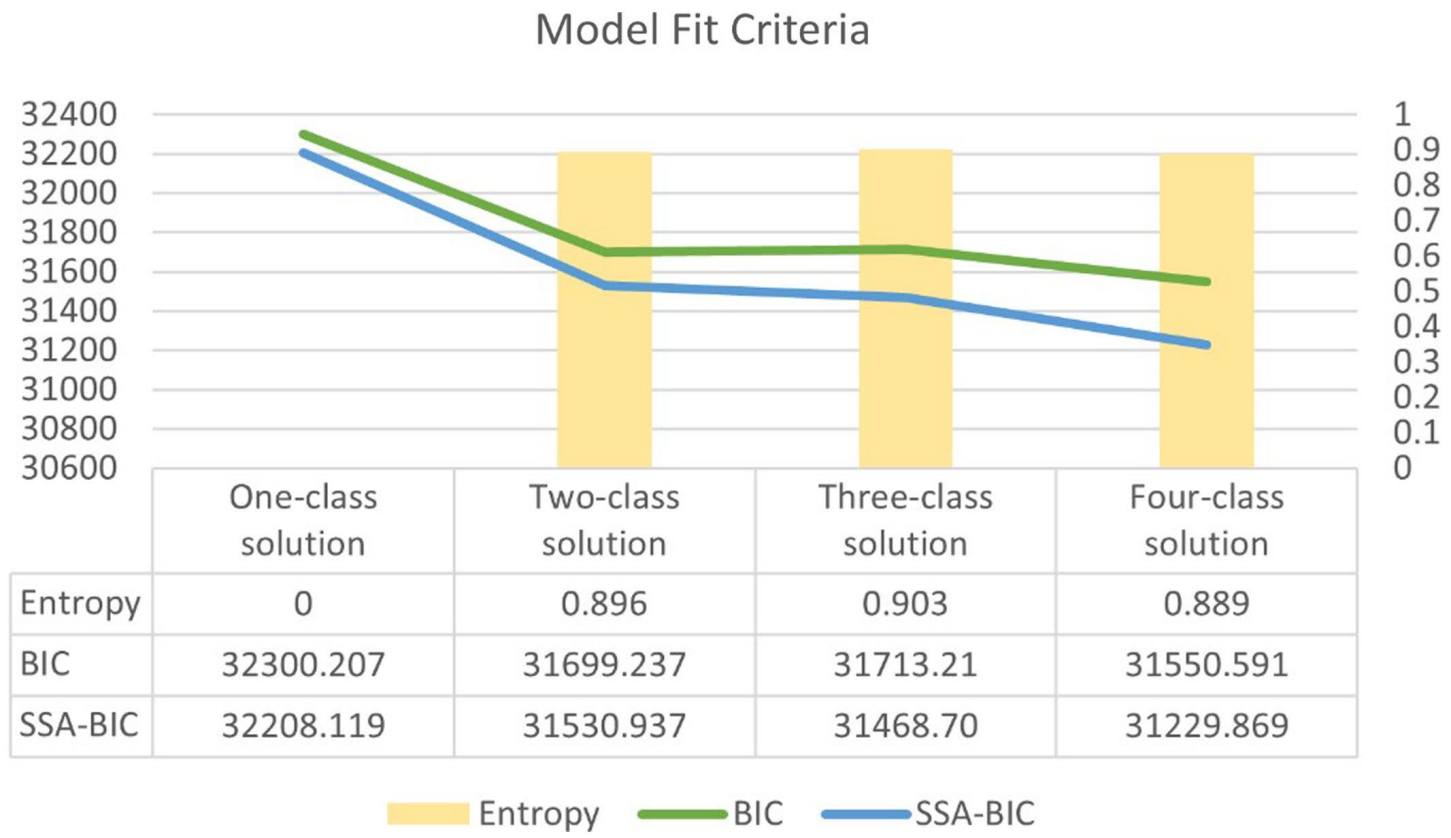
Latent class model fit criteria comparison.

The four-class solution was selected for interpretation and had an entropy of 0.889 (see Figure 2). Profiles or classes were named based on the distal outcome of cardiometabolic disease as follows: Class 1 represents the lowest risk, Class 2 indicates modest risk, Class 3 reflects moderate risk, and Class 4 signifies the highest risk.

Class 1, representing the *lowest risk profile* (73.8% of sample), exhibited the highest conditional probabilities of not having hypertension or diabetes, at 53.9 and 45.6%, respectively. Additionally, this class the lowest mean BMI of 25.2 kg/m^2^ compared to all other classes. Class 1 also had the highest conditional probabilities of being in the US for more than 20 years (71.3%), speaking English well or very well (92.5%), eating South Asian foods equally with other food types at home (42.9%), having a high desire to pass down cultural traditions (30.8%), and receiving neighborhood support most to all of the time (82.8%). In addition, this group had the lowest mean scores for R/S struggles and positive spiritual coping scores (1.2 and 2.5, respectively).

Class 2, representing the *modest risk profile* (13.4% of sample), had a conditional probability of no hypertension of 50.9%, with a mean BMI of 26.0 kg/m^2^. Although this class had the second lowest conditional probability of diabetes (20.8%), it exhibited the highest conditional probability of prediabetes (35.7%). Additionally, Class 2 had the highest conditional probabilities of eating food at home that was mostly not South Asian or only other non-South Asian cuisine (10.3%), some desire to pass down cultural traditions (61.3%), little to no social support (26.6%), friends who were predominantly non-South Asian or exclusively from other non-South Asian backgrounds (17.8%), and experienced daily to weekly discrimination (96.9%). Class 2 had the highest mean scores for depressive symptoms, and trait anxiety and anger (19.7, 22.4, and 18.9, respectively), indicating significant depressive symptomology, as well as high trait anxiety and moderate trait anger.

Class 3, representing the *moderate risk profile* (8.9% of sample), had the second highest conditional probabilities of reporting hypertension (55.9%) and diabetes (38.4%) with a mean BMI of 25.9 kg/m^2^. Class 3 exhibited the highest likelihood of having resided in the US less than 10 years (13.6%), having limited English proficiency (10.2%, not at all, or fairly well, 23.5%), predominately consuming South Asian cuisine at home (72.5%), showing minimal interest in passing down cultural traditions (43.3%), receiving occasional social support (20.8%), and maintaining predominantly South Asian friends (64.2%). Additionally, this group had demonstrated the second highest mean scores for depressive symptoms, and trait anxiety and anger (9.1, 16.6, and 16.2, respectively), indicating moderate levels of trait anxiety and anger within this profile. Class 3 also had the highest mean score for R/S struggles and positive spiritual coping (2.7 and 3.5, respectively).

Class 4, representing the *highest risk profile* (3.9% of sample), exhibited the highest conditional probabilities of reporting hypertension (84.2%) and diabetes (45.4%), as well as the highest mean BMI of 36.4 kg/m^2^. Class 4 was characterized by significant likelihood of residing in the US for 10 to 20 years (45.8%), receiving consistent social support (92.4%), but limited neighborhood support (34.2%). Additionally, individuals in Class 4 were equally likely to have friends from both South Asian and other race/ethnic backgrounds (48.1%). This group had the second highest probability of expressing little to no desire to pass down cultural traditions (31.5%). Despite these risk factors, Class 4 exhibited the lowest mean scores for trait anxiety and anger, as well as depressive symptoms (14.3, 14.6, and 4.5, respectively). Class 4 had the second lowest mean score for R/S struggles and third highest score for positive spiritual coping (i.e., 1.3 and 2.9, respectively) (see Table 3 for further details).

**Table 3:** Four-class solution conditional probabilities and means for acculturation profiles and distal outcome of cardiometabolic risk (*N* = 771).

	Class 1	Class 2	Class 3	Class 4
	Lowest risk	Modest risk	Moderate risk	Highest risk
	569	103	69	30
	73.8%	13.4%	8.9%	3.9%
Hypertension				
No	0.539	0.509	0.441	0.158
Yes	0.461	0.491	0.559	0.842
Diabetes status				
No	0.456	0.436	0.306	0.221
Pre-diabetic	0.329	0.357	0.310	0.326
Diabetic	0.215	0.208	0.384	0.454
BMI Asian cat.	25.2	26.0	25.9	36.4
Years in the US				
<10 years	0.052	0.059	0.136	0.000
10–20 years	0.235	0.278	0.185	0.458
>20 years	0.713	0.663	0.678	0.542
Speaking English				
Not at all or poorly	0.018	0.044	0.102	0.041
Fairly well	0.057	0.133	0.235	0.085
Well or very well	0.925	0.823	0.663	0.873
Food eaten at home				
Only or mostly S. Asian	0.495	0.552	0.725	0.523
Equally S. Asian or other	0.429	0.345	0.245	0.413
Mostly or only other	0.075	0.103	0.030	0.064
Desire to pass down cultural traditions				
Little to none	0.138	0.210	0.433	0.315
Some	0.554	0.613	0.516	0.497
High	0.308	0.177	0.051	0.188
Social support				
Little to none	0.026	0.266	0.053	0.000
Some of the time	0.158	0.370	0.208	0.076
Most to all of the time	0.817	0.364	0.739	0.924
Neighborhood support				
Little to none	0.172	0.235	0.251	0.342
Most to all of the time	0.828	0.765	0.749	0.658
Friends				
Only or mostly S. Asian	0.531	0.535	0.642	0.486
Equally S. Asian or other	0.366	0.287	0.343	0.481
Mostly or only other	0.103	0.178	0.014	0.033
Discrimination				
Rarely to never	0.210	0.031	0.229	0.218
Daily to weekly	0.790	0.969	0.771	0.782
CES-D scale score	4.9	19.7	9.1	4.5
Spielberger trait anxiety score	14.7	22.4	16.6	14.3
Spielberger trait anger score	15.4	18.9	16.2	14.6
Religious/spiritual struggles score	1.2	1.5	2.7	1.3
Positive spiritual coping score	2.5	2.8	3.5	2.9

### Covariates for latent class membership

Our auxiliary multinomial logistic regression revealed the following. When considering social and environmental determinants of health, we found that individuals in the modest risk profile (Class 2) had decreased odds of earning an income of $75,000 or more compared to those in the lowest risk profile (Class 1). Additionally, those in the moderate risk profile (Class 3) exhibited decreased odds of having a bachelor’s degree or higher compared to those in the lowest risk profile (Class 1). Among health behavior covariates, individuals in moderate risk profile (Class 3) were less likely to be never smokers compared to the lowest risk profile (Class 1). Regarding religiosity and spirituality behaviors and attitudes, individuals in the modest risk profile (Class 2) and moderate risk profile (Class 3) were associated with increased odds of finding it hard to forgive themselves compared to those in the lowest risk profile (Class 1). Furthermore, individuals in the modest risk profile (Class 2) showed decreased odds of experiencing deep inner peace/harmony compared to those in the lowest risk profile (Class 1). Finally, individuals in the moderate risk profile (Class 3) were associated with increased odds of expressing gratitude to [God] in daily life and frequently attending religious services compared to those in the lowest risk profile (Class 1) (see Table 4 for further details).

**Table 4:** Auxiliary multinomial logistic regression of sociodemographic factors, social and environmental determinants of health, health behaviors, and religiosity and spirituality covariates on the four-class solution of cardiometabolic risk profiles using Class 1 or lowest risk profile as reference (*N* = 552).

	Modest risk			Moderate risk			Highest risk		
		95% CI			95% CI			95% CI	
	OR	Lower	Upper	OR	Lower	Upper	OR	Lower	Upper
55 years and older	0.72	0.34	1.54	2.27	0.97	5.34	0.34	0.08	1.49
Income $75,000 or over	**0.31**	**0.14**	**0.67**	**0.38**	**0.16**	**0.89**	1.19	0.27	5.24
Bachelor’s degree or above	0.51	0.17	1.49	**0.23**	**0.07**	**0.80**	0.39	0.07	2.08
Female	1.72	0.80	3.71	1.66	0.67	4.09	0.72	0.20	2.64
San Francisco, CA (Chicago, IL as ref.)	0.93	0.43	2.00	0.87	0.38	1.95	0.88	0.30	2.64
High physical activity	0.56	0.26	1.22	1.32	0.57	3.06	0.94	0.27	3.24
Frequent yoga	1.11	0.55	2.21	0.63	0.28	1.42	1.03	0.34	3.15
Never smoker	0.41	0.15	1.13	**0.25**	**0.08**	**0.76**	0.54	0.11	2.63
Any alcohol use	1.12	0.50	2.47	0.51	0.18	1.42	1.51	0.38	6.01
Eat out frequently	1.58	0.93	2.68	0.79	0.40	1.52	1.03	0.45	2.36
Hindu (religious tradition)	0.52	0.21	1.26	1.91	0.83	4.37	0.86	0.24	3.07
Find it hard to forgive myself?	**3.10**	**1.31**	**7.33**	**3.95**	**1.47**	**10.6**	2.40	0.70	8.20
Others have not forgiven me…	1.57	0.74	3.32	1.84	0.79	4.31	2.25	0.76	6.67
I feel deep inner peace/harmony	**0.25**	**0.11**	**0.59**	1.23	0.55	2.74	1.26	0.26	6.22
Experience connection to all life	0.86	0.40	1.85	0.90	0.44	1.86	0.96	0.22	4.24
Spirituality is about personal rel. w/[God]	7.17	0.67	77.3	0.75	0.25	2.26	1.62	0.45	5.77
Express gratitude to [God] in daily life	1.09	0.38	3.09	**8.73**	**1.45**	**52.6**	1.50	0.27	8.23
Frequent attendance to religious services	0.66	0.26	1.64	**4.51**	**1.60**	**12.7**	0.89	0.23	3.52

Bold value means significant findings based on 95% confidence intervals.

## Discussion

**Figure 3:**
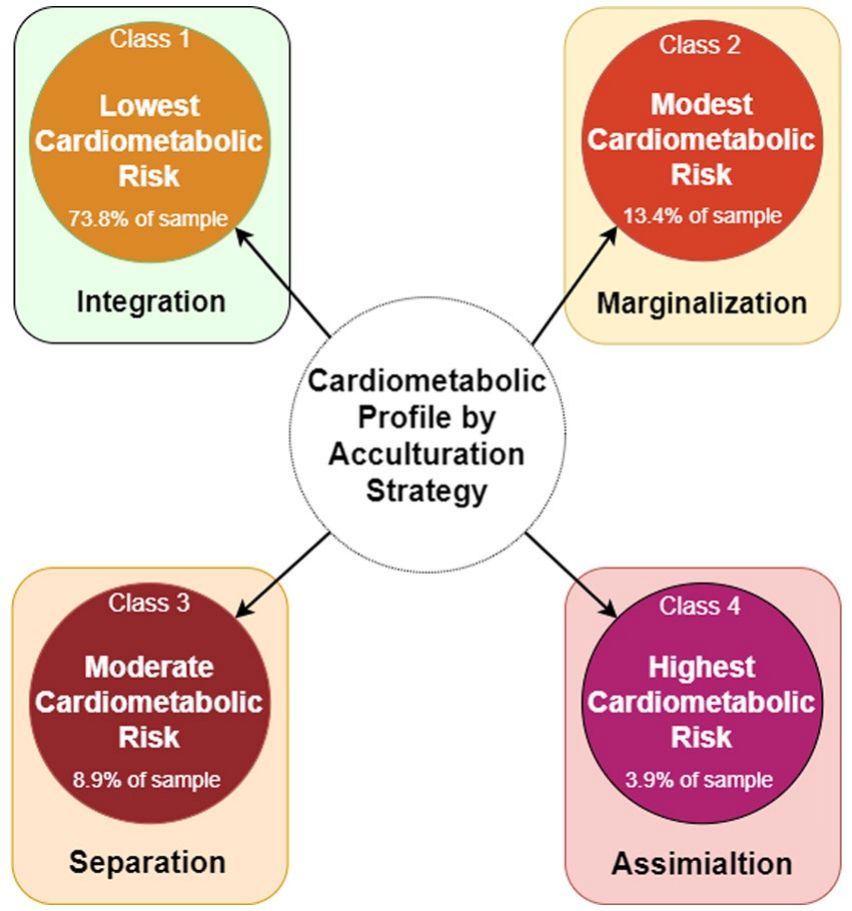
Profiles of cardiometabolic risk classified by probable acculturation strategy.

**Figure 4:**
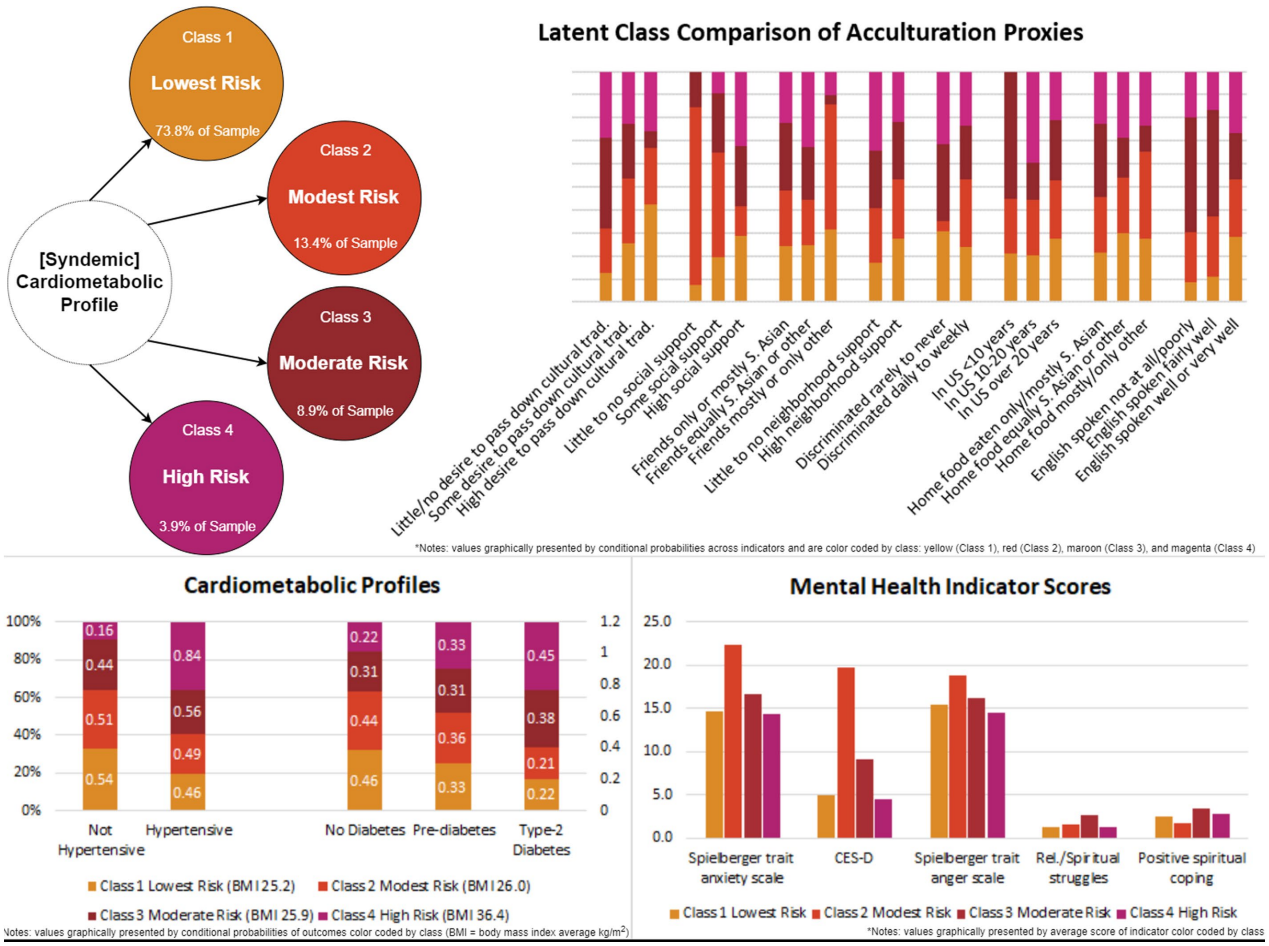
Comparison of identified profiles using conditional probabilities by acculturation and acculturative stress proxies and cardiometabolic conditions.

We identified four nuanced cardiometabolic risk profiles among South Asians using indicators of acculturation (see Figure 3 for visualization of the identified profiles of cardiometabolic risk classified by probable acculturation strategy and Figure 4 for a comparison of these profiles). Although we identified a lowest risk group, this profile was found to be on the cusp of cardiometabolic disease. On average, individuals in this profile were overweight, with a significant conditional probability of hypertension and prediabetes, which could progress to cardiometabolic syndrome. Each profile revealed key indicators for cardiometabolic syndrome and acculturation, allowing us to identify points of risk and intervention. Furthermore, we identified possible acculturation strategies by utilizing Berry’s model of acculturation and Needham et al. (31) prior findings. We classified each risk profile as possibly utilizing the following strategies of integrated, separated, assimilated, or marginalized. We also incorporated aspects of biculturalism from LaFromboise et al. (33) model of acculturation to account for possible alternation or strategic movement between the South Asian individual’s and US host culture. Incorporating alternation allows for greater complexity in our identified acculturation models, enabling exploration or how indicators may function as possible strategies affecting cardiometabolic outcomes. For example, Class 1, or the lowest risk profile, could be considered integrated, as individuals in this group maintain their cultural identity while fully participating in US host culture. This profile is characterized by the highest likelihood of residing in the US more than 20 years, proficiency in English, equal consumption of South Asian and other cuisines at home, and a strong desire to preserve cultural traditions, compared to other profiles. Additionally, individuals in this profile have a high likelihood of having friends from diverse backgrounds, along with high levels of social support and neighborhood support. Of note is that neighborhood environment may play a protective role in profile as evidenced by the highest neighborhood support conditional probability compared to other profiles. For instance, Lagisetty et al. (19) reported that South Asian women from the MASALA cohort experienced a 46% reduced odds of hypertension when residing in neighborhoods with high support. From a bicultural framework, we argue that enculturation is also occurring, involving the reintegration or relearning of an individual’s native culture as an adaptive strategy (62). This is supported by the fact that individuals in this profile exhibited the lowest mean scored for R/S struggles, as well as the second lowest scores for depressive symptom, and trait anxiety and anger. The overall bicultural indicators and enculturation strategy may help explain why this profile had the lowest likelihood of hypertension, diabetes, and high BMI, despite technically falling into the overweight category according to Asian BMI classifications. The likelihood of prediabetes remained high, however, indicating increased metabolic risk in the lowest risk profile or Class 1.

A possible indictor that may provide some context in elevating cardiometabolic risk is discrimination. All identified profiles had high likelihoods of reporting perceived discrimination, ranging from 77 to 97%. This could account for the elevated scores in depressive symptoms, elevated scores in and trait anxiety and anger. Previous studies have reported higher levels of discrimination, which were associated with poorer mental health outcomes among US South Asians (37). Discrimination has long been linked to numerous health and mental health disparities among African Americans and Hispanics/Latinos (63, 64). The relationship is less clear among US South Asians, however, as several studies have found no direct association between discrimination and cardiovascular health (36, 37). Discrimination does not appear to be indicative of food choice and consumption. Another potential factor of interest may be low social support, as US South Asians with low social cohesion have been found to have higher likelihood of increased BMI and hypertension (19, 20). Nonetheless, discrimination was most prevalent in the modest risk profile or Class 2.

The modest risk profile exhibited a higher likelihood of reporting hypertension compared to Class 1, indicating a hypertensive risk within this subgroup. Additionally, while this profile had the second largest likelihood of not having diabetes, it had the highest likelihood of prediabetes among all other profiles. Furthermore, individuals in this profile demonstrated the highest likelihood of expressing some desire to pass down cultural traditions, as well as consuming non-South Asian foods at home and having friends who were not South Asian descent. In addition, this profile displayed the highest scores for depressive symptom, as well as trait anxiety and anger scores, suggesting a high-level mental health distress and acculturative stress. Individuals in the modest risk profile also exhibited the lowest likelihood of receiving social support and the highest likelihood of experiencing discrimination. Therefore, despite having modest cardiometabolic risk factors, Class 2 showed elevated mental health distress and may likely be employing a marginalization strategy. Marginalization acculturation strategies often involve the rejection of both their culture and that of the host culture, which is often associated with high levels of discrimination (27). This marginalization strategy may help explain the observed distribution of low to moderate desires to pass on cultural traditions, low social support, along with a high likelihood of maintaining friendships predominantly with non-South Asian individuals. Failed attempts at integrating into the host culture can result in loss of the individual’s cultural identity and contribute to increased mental health issues (27), as evidenced in this profile. The buffering effect against cardiometabolic risk factors in the modest risk profile may be attributed to their moderate levels positive spiritual coping. However, the moderate score for positive spiritual coping within this class may exacerbate the effects of R/S struggles, coinciding with increased trait anxiety and anger (65), as well as depressive symptoms (66).

Studies focusing on health, acculturation and spirituality are limited, especially in US South Asians. Positive spiritual coping has been associated with better self-rated health and emotional wellbeing among South Asians (46). Religious struggle may exacerbate the effects of acculturative stress, as measured by psychosocial factors. A study by Tobin and Slatcher (67) found that religious struggles mediate the relationship between religious participation and healthy cortisol slopes. Cortisol, a hormone that helps regulate metabolism among other physiological function, may be influenced by these struggles. It is possible that positive spiritual coping may be offset cardiometabolic syndrome and other potential positive health effects (43, 46). Additionally, a study by Ai et al. (65) found that spiritual struggles mediated the effects of anxiety and anger on interleukin-6—a biomarker of inflammation associated with cardiometabolic disorders (68)—among patients. Among these patients, interleukin-6 mediated the effects on hostility after surgery and counteracted the effects of positive religious coping (65). A possible focus for future studies will be on the interaction of acculturation and R/S directly. Not unlike a study among US Vietnamese, Luu et al. (54) found that high levels of acculturation and lower levels of spirituality increased positive and favorable attitudes for help seeking. All of the aforementioned R/S factors in conjunction with biomarkers may help explain why the modest risk profile is at the cusp of possible cardiometabolic syndrome. Although both R/S struggles and positive spiritual coping were the highest in this profile compared to all others, more is needed to disentangle the possible protective factors each offers in the acculturation and cardiometabolic risk relationship. Notably, the progenitor of the R/S struggles scale has stated that religious struggles should not be considered entirely maladaptive. Rather, they are influenced by the “interplay between personal, situational, and social-cultural factors, as well as by the way in which health and well-being are conceptualized and measured” (69). The moderate risk profile represents a point of interventional focus and further exploration for preventive programs.

Class 3 or the moderate risk profile, had the highest likelihoods of living in the US less than 10 years and spoke English from not at all to fairly well, although the proportion was small. The profile also had the second highest mean scores for depressive symptoms and trait anxiety and anger. Social and neighborhood support were not the highest, suggesting that the built environment might not have been conducive to integration with the host or South Asian community. The moderate risk profile could be argued to employ a separation strategy due to having the lowest likelihood of spoken English proficiency, as well as the highest likelihood of having friends and eating cuisine at home that was almost exclusively South Asian. Alternatively, their little to no desire to pass down cultural traditions may also indicate a marginalization strategy. Alternation may at play in this profile where individuals are moving to and from between South Asian and US culture. The moderate risk profile may illustrate a situation where individuals are in a liminal phase or an ‘in between’ separation, marginalization, or assimilation process as traditional cultural traditions may be weathering in the presence of discrimination with low linguistic integration while trying to maintain similar peer groups and social support. This liminality may be indicated by the high levels of mental health distress. The higher likelihoods of social and neighborhood support, as well as having the highest spiritual coping and struggles scores, may be buffer the depressive symptomology that was observed in the Class 2 modest risk profile. Alternatively, this profile may be alternating acculturation strategies with separation, marginalization, and assimilation where R/S struggles and coping may be indicating a possible interaction with self and identity between South Asian and US host culture. Overall, the moderate cardiometabolic risk profile may indicate an assimilation trajectory where the individual gives up their own cultural identity and becomes absorbed into the host culture.

While Class 4 represented the smallest subgroup, it exhibited the highest risk profile as multiple biomarkers suggestive of possible cardiometabolic syndrome. This profile demonstrated the highest conditional probabilities hypertension, type 2 diabetes, and obesity. Individuals within this subgroup were most likely to have resided in the US between 10 and 20 years, reported the lowest level of neighborhood support, and showed the second highest likelihood of expressing little to no desire of passing down cultural traditions. Furthermore, the highest risk profile displayed the second lowest score for R/S struggles score and second highest score for positive coping. Interestingly, despite being the highest risk profile, individuals in this class exhibited the lowest CES-D scores, as well as lowest mean scores for trait anxiety and anger, compared to all other classes. The profile presents a paradoxical relationship between mental health and cardiometabolic outcomes. Moreover, dietary habits at home showed similar conditional probabilities to those of the lowest risk profile. One could argue for an assimilation strategy within this risk profile, given the high likelihoods of proficiency in English, consumption of both South Asian and other race/ethnicity foods at home, and reduced desire to pass down cultural traditions.

To further identify and examine nuanced differences between profiles, we examined covariates of risk not associated with acculturation from the social and environmental determinants of health, health behaviors, and religiosity/spirituality using an auxiliary approach to the LCA. We found low income to be the most pronounced determinant of health between the modest and moderate risk profiles compared to lowest risk profile. Additionally, the moderate risk subgroup also had much lower odds of having a bachelor’s degree. It is worth noting that South Asians tend to be among the highest SES immigrant groups with higher levels of income and education when compared to other racial and ethnic groups (70). In terms of health behaviors, the moderate risk profile had lower odds of being a never smoker. We also assessed the role of R/S behavior and attitude covariates between classes given their relationship with R/S coping, acculturation, and cardiometabolic risk to further differentiate our identified risk profiles. When comparing our lowest risk profile to the moderate risk profile with highest mean positive coping and R/S struggles scores, the moderate profile reported higher odds of forgiving themselves. This was similar to the modest risk profile, which had higher likelihood of mental health distress, reporting over 200% odds of finding it hard to forgive themselves, while also struggling to feel any deep inner peace or harmony when compared to the lowest risk profile. Expressions of gratitude to God or the divine in daily life were a major factor of differentiation between the modest and moderate risk profiles, as well as frequent religious services attendance. The lower mental health distress scores, particularly with lower anxiety, may be associated with expressions of gratitude to God or the divine as a previous findings have suggested (46), but may not have been the lowest as frequent services attendance has been associated with increased levels of anxiety (51). The role of spiritual coping and R/S in acculturation and cardiometabolic risk needs further exploration.

Overall, the lowest risk profile and the highest risk profile were observed to have nuanced differences in social aspects of the acculturative process. While slight differences in conditional probabilities of high social support were observed between the lowest and highest risk profiles, pronounced differences were noted in received neighborhood support and types of friends kept. Furthermore, while social covariates did not differentiate between these risk profiles, major differences can be found in length of years living in the US, the desire to pass down cultural traditions, and neighborhood support. More years lived in the US have been associated with higher risk, yet when compared to the lowest risk profile, the highest risk profile had a lower likelihood of having lived more than 20 years in the US (25). A desire to pass down cultural traditions and neighborhood support must then play a key role, as they have been found to have a positive influence in health outcomes, potentially driving differences in risk between profiles (19, 25). Perhaps minor differences in food eaten at home, where dietary patterns follow a more metabolically risky trajectory, also contribute (39–41). While the highest risk profile has higher social support, their social network could be enabling larger body size norms (22). The differences between profiles are complex, but these nuances may reveal that points of interaction that may exponentially increase risk. For instance, the observed differences in conditional probabilities of neighborhood support and friends warrant further exploration to assess the adaptive and maladaptive role of these indicators on cardiometabolic risk. Regardless of the mutually exclusive nature of latent classes, the interactions between indictors, irrespective of the differences in conditional probabilities observed, help differentiate cardiometabolic profiles and promote the contextual exploration of identified profiles and risk.

Nuanced differences in acculturation indicators identify not only highlight syndemic cardiometabolic risk and mental health distress but also underscore the role of acculturation strategies (see Figure 3). For example, the moderate and modest cardiometabolic risk profiles exhibit the highest mean scores for depressive symptoms, trait anxiety, and trait anger scores, respectively. In the modest cardiometabolic risk profile, the adoption of marginalization acculturation strategy may signal heightened mental health distress and potential risks to developing hypertension and type 2 diabetes. This profile underscores the possible intertwined relationship between mental health and cardiometabolic disease. Conversely, within the moderate risk profile, despite showing the second highest likelihoods of mental health distress, there may be indications of a liminal group and acculturation process. For example, while the moderate and highest risk profiles may align with an assimilation strategy, subtle differences in social interactions and dietary habits could suggest shifts toward other strategies such as separation or marginalization within the moderate profile. These variations emerge as individuals navigate their environment and are shaped by socioecological factors, including experiences of discrimination. Consequently, South Asians may lean toward marginalization, lading to heightened mental health challenges compared to those following integration, separation, or assimilation strategies. The moderate cardiometabolic risk group might signify a transitional phase, transitioning between acculturation strategies and warranting further exploration. This in-between status of the moderate cardiometabolic risk subgroup may represent a critical transitional phase, necessitating further exploration to identify appropriate interventions for mitigating for both cardiometabolic health and mental health risks. Understanding the role of place and neighborhood becomes crucial in elucidating cardiometabolic risk factors and potential buffers the burden of disease. Future research will delve into longitudinal effects of acculturation on cardiometabolic disease, offering insights into immigrant health within the context of transitional risk.

### Limitations

Our study is among the first to identify cardiometabolic risk profiles that incorporate a complex construct of acculturation while assessing covariates not included in the model to predict latent class membership, including social determinants of health, health behaviors, and religion and spirituality. However, our study, was not without limitations. First, our analysis was cross-sectional and examined profiles from one time point capturing a person-centered context but not the dynamic changes associated with the acculturative process. Thus, we could not determine the direction of associations or changes in cardiometabolic risk and acculturation over time. Future studies will use longitudinal data to explore transitions between cardiometabolic risk profiles, enhancing our understanding of dynamic acculturation.

Second, some data were self-reported, making them susceptible to biases such as projection, reporting, or recall biases, including “white coat” bias. Third, our sample was primarily composed of Asian Indians, who tend to have higher SES and education levels compared to other South Asian subgroups, such as Bhutanese immigrants and refugees. Finally, using Berry’s model of acculturation, we linearized the process, which may oversimplify its complexity. Despite this, Berry’s model facilitates categorization. Incorporating biculturalism aspects from LaFromboise et al. (33) model allowed us to account for the strategic alternation between the individual’s heritage and host cultures.

## Conclusion

While previous studies have focused on the linear and variable-centered relationships of acculturation and cardiovascular risk among South Asians in the US, we have taken a person-centered approach to identify risk profiles in context of multiple acculturation factors and acculturative stress on the syndemic outcomes of cardiometabolic disease. We identified four cardiometabolic risk profiles among US South Asians (see Figure 4 for a graphical summary), enhancing our understanding of their disparate health outcomes within a person-centered context. Our study also expands complex acculturation models for rapidly growing immigrant groups like US South Asians, and highlight the need to holistically incorporate interacting processes that synergize negative outcomes. Our comprehensive and transdisciplinary model has and will enable the development of more nuanced and inclusive measures to better encapsulate acculturation as a dynamic process and its role in health and disease among US South Asian immigrant subgroups. Overall, our model and methodology, facilitates a holistic but nuanced exploration of the acculturation process and its interacting risk and protective factors that can enable the development of culturally sensitive, tailored interventions to promote overall well-being among US South Asians.

## Data Availability

The data analyzed in this study is subject to the following licenses/restrictions: data used for this analysis are restricted but can be made available upon request to the MASALA (Mediators of Atherosclerosis in South Asians Living in America) program via application process. Requests to access these datasets should be directed to https://www.masalastudy.org/for-researchers.

## Footnotes

^1^https://www.masalastudy.org/for-researchers
